# Annual Incidence Rate of Infectious Diseases Estimated from Sentinel Surveillance Data in Japan

**DOI:** 10.2188/jea.13.136

**Published:** 2007-11-30

**Authors:** Shuji Hashimoto, Yoshitaka Murakami, Kiyosu Taniguchi, Nahoko Shindo, Ken Osaka, Hiroshi Fuchigami, Masaki Nagai

**Affiliations:** 1 Department of Hygiene, Fujita Health University School of Medicine.; 2 Epidemiology and International Health Research Section, Environmental Health Sciences Division, National Institute for Environmental Studies.; 3 Infectious Disease Surveillance Center, National Institute of Infectious Diseases.; 4 Department of Public Health, Saitama Medical School.

**Keywords:** incidence, infectious disease, surveillance, influenza-like illness

## Abstract

BACKGROUND: The estimation of incidence rates of infectious diseases based on the sentinel surveillance data is rather rare. We attempted to estimate these in 2000 in Japan by the surveillance data, and to evaluate their biases.

METHODS: We used the incidences of influenza-like illness and 12 pediatric diseases in each of the sentinel medical institutions in Japan based on surveillance data in 2000. The incidence in all medical institutions was estimated under the assumption that the sentinel medical institutions were randomly selected. The possible bias of this estimate was evaluated in comparison with the hypothetical true incidence obtained as the total incidence in all medical institutions estimated by a regression model using the numbers of all disease outpatients per day from the National Survey of Medical Care Institutions of Japan.

RESULTS: The estimated annual incidence rate was 75.6 (95% confidence interval: 72.3-78.7) per 1,000 population in influenza-like illness, and ranged from 1.1 (95% confidence interval: 1.0-1.2) to 285.2 (95% confidence interval: 270.2-300.3) per 1,000 population aged 0-19 years among 12 pediatric diseases. The ratio of the estimated incidence to the hypothetical true one was 1.06-1.26 among influenza-like illness and the 12 pediatric diseases.

CONCLUSIONS: The incidence rates of influenza-like illness and pediatric diseases in 2000 in Japan were estimated from sentinel surveillance data. The rates obtained provide some useful but not always accurate information. Thus, further research is necessary.

The surveillance of infectious diseases has been established in many countries.^1^^-^^10^ The estimation of the incidence rates of infectious diseases based on the sentinel surveillance data is rather rare, although such surveillance provides some useful information regarding incidence.^11^^-^^13^ In many surveillance projects including one in Japan, sentinel medical institutions (SMIs) are recruited on a voluntary basis. However, uncertainties remain as to how representative the finding are when applied to all the medical institutions in given areas. In the method for estimating incidence rates by surveillance data, it would be assumed that SMIs are randomly selected from all the medical institutions if information on the underlying SMI population is not available.^11^^,^^14^

In Japan, the guidelines for the surveillance, introduced in 1999 by the Ministry of Health, Labor and Welfare, determine that SMIs are selected from all medical institutions in the areas as randomly and as representatively as possible (http://idsc.nih.go.jp/index.html).^15^ Prefectural governments select SMIs according to the guidelines provided. The numbers of all disease outpatients per day in each medical institution from the National Survey of Medical Care Institutions conducted by the Ministry^16^^,^^17^ are available and would be useful to evaluate the underlying SMI population.

We attempted to estimate the incidence rates of infectious diseases in 2000 in Japan from surveillance data under the assumption that SMIs were randomly selected from all medical institutions. We also sought to evaluate the possible bias in these estimated incidence rates using the number of all disease outpatients per day in each medical institution.

## MATERIALS AND METHODS

### Surveillance of infectious diseases in Japan

The National Epidemiological Surveillance of Infectious Diseases in Japan is organized by the Ministry of Health, Labor and Welfare.^9^^,^^10^ It involves the systems for influenza-like illness and pediatric diseases. Local governments select SMIs according to the Ministry guidelines. The numbers of SMIs in the areas covered by public health centers are approximately proportional to their population sizes. In reality, SMIs seem to be recruited on a voluntary basis to some extent, although the Ministry guidelines call for the SMIs to be selected from all the medical institutions in the areas as randomly and as representatively as possible.^15^ Each SMI reports the numbers of cases of notifiable infectious diseases to the area public health center weekly. Notification by public health centers to the local government and the Ministry is made through an on-line computer network.

### Surveillance data and method for estimating incidence rates

Table 1 shows the numbers of all medical institutions and SMIs in 2000. About 94% of SMIs reported all 52 weeks in 2000, and the others started or stopped reporting in 2000. The pediatric disease surveillance system included 3,011 SMIs, which were departments of pediatrics in hospitals and clinics with a department of pediatrics. The influenza-like illness surveillance system included 4,656 SMIs, which were departments of internal medicine or pediatrics in hospitals and clinics with departments of internal medicine and/or pediatrics. Those included most SMIs for the pediatric disease surveillance system. Proportions of SMIs among all medical institutions ranged from 1.3% in clinics with a department of internal medicine and without a department of pediatrics, to 30% or more in clinics with a department of pediatrics and without a department of internal medicine. The numbers of all medical institutions were obtained from the National Survey of Medical Care Institutions conducted by the Ministry in October 1999.^16^

**Table 1.** The numbers of all and sentinel medical institutions.

	Department	No. of all medical institutions	No. of sentinel medical institutions			

			for Influenza-like illness		for Pediatric diseases	
Hospital	Internal Medicine	8,130	457	5.6%		
	Pediatrics	3,575	730	20.4%	734	20.5%
Clinic	Internal Medicine only	37,454	470	1.3%		
	Pediatrics only	3,411	1,155	33.9%	1,145	33.6%
	Internal Medicine and Pediatrics	23,842	1,844	7.7%	1,132	4.7%
Total		76,412	4,656		3,011	

The percentages are in relation to the number of all medical institutions.

We used 238,201 reports from the influenza-like illness surveillance system and 155,184 reports from the pediatric disease surveillance system. These reports covered about 98% observations over 52 weeks in each of the SMIs in 2000. Each report included the sex- and age-specific numbers of influenza-like illness patients newly diagnosed during a given week in the influenza-like illness surveillance system and the numbers of patients aged 0-19 years with 12 pediatric diseases (see Table 3) in the pediatric disease surveillance system. In each SMI, the sex- and age-specific annual incidences of influenza-like illness and the annual incidences of each pediatric disease in the patients aged 0-19 years were calculated as the totals of those in weekly reports. The missing data (less than 2% among the total reports) were replaced with the mean incidence calculated for each disease, week, prefecture and type of medical institution (specifically also for each sex and age group in influenza-like illness). The medical institutions for pediatric diseases were classified into 3 types; departments of pediatrics in hospitals, clinics with only a department of pediatrics and clinics with departments of internal medicine and pediatrics. Those for influenza-like illness were classified into four types, the above three types and others, because the number of SMIs in either the departments of internal medicine in hospitals or clinics with only a department of internal medicine was 1 or less in several prefectures.

For each disease, prefecture, and type of medical institution, the incidences in SMIs follow a multi-hypergeometric distribution under the fixed condition of the total number of SMIs under the assumption that SMIs are randomly selected from all medical institutions. The total incidence in each prefecture and type of medical institution was estimated as the total incidence in SMIs divided by the proportion of SMIs to all medical institutions. The total incidence in all medical institutions was estimated to be the total of those in all prefectures and types of medical institution. The approximate confidence interval for the incidence was given based on the distribution. The appendix shows the method for estimating incidences in detail.

### Method for evaluating biases of estimated incidence rates

We used the numbers of all disease outpatients in September 1999 in each department of internal medicine and pediatrics in hospitals and those over one week in September 1999 in each clinic from the National Survey of Medical Care Institutions of Japan.^16^ The number of all disease outpatients per day was used in 4,585 out of 4,656 SMIs and in 76,412 of all medical institutions for estimating the incidence of influenza-like illness, and in 2,991 out of 3,011 SMIs and 30,828 of all medical institutions for estimating the incidences of pediatric diseases.

For each disease and type of medical institution, a linear regression model with a dependent variable for the incidence and independent variables for the number of all disease outpatients per day and prefecture (as dummy variables) was applied using the SMI data. The hypothetical incidence in every medical institution was calculated as the expected value using an estimated linear regression equation and the data of its independent variables. Using the hypothetical incidences in all SMIs, the incidence in all medical institutions was estimated by the above-explained method. The bias of this estimate was evaluated in comparison with the hypothetical true incidence obtained as the total hypothetical incidence in all medical institutions.

## RESULTS

Table 2 shows the estimated incidence of influenza-like illness in 2000 in Japan. The annual incidence was estimated to be 9,590,000. The annual incidence rate per 1,000 population was estimated to be 75.6 (95% confidence interval [CI]: 72.3-78.7). The rate was slightly higher in males than in females, and decreased with age.

**Table 2.** Estimated incidences of influenza-like illness.

		Incidence	Incidence rate	95% confidence interval
Total		9,590,000	75.6	72.3-78.7

Sex	Males	4,900,000	78.9	75.7-82.3
	Females	4,680,000	72.2	69.1-75.4

Age	0-4 years	1,550,000	262.5	247.3-276.1
	5-9	2,000,000	332.1	317.2-348.7
	10-14	1,040,000	158.9	151.2-166.5
	15-19	720,000	96.2	90.8-101.5
	20-29	1,110,000	60.9	57.1-64.2
	30-39	1,130,000	66.9	62.8-70.4
	40-49	720,000	43.1	40.7-46.1
	50-59	600,000	31.3	29.2-33.9
	60-69	410,000	27.6	25.6-30.3
	70-	310,000	20.8	18.8-22.8

The incidence rate and its 95% confidence interval are stated per 1,000 population.

Table 3 shows the estimated incidences of 12 pediatric diseases in 2000 in Japan. The annual incidence in population aged 0-19 years ranged from 28,000 in pertussis to 7,405,000 in infectious gastroenteritis. The annual incidence rate per 1,000 population aged 0-19 years ranged from 1.1 (95% CI: 1.0-1.2) to 285.2 (95% CI: 270.2-300.3).

**Table 3.** Estimated incidences of pediatric diseases..

Pediatric disease	Incidence	Incidence rate	95% confidence interval
Pharyngoconjunctival fever	171,000	6.6	5.7-7.4
Group A streptococcal pharyngitis	1,286,000	49.5	45.8-53.3
Infectious gastroenteritis	7,405,000	285.2	270.2-300.3
Chickenpox	2,344,000	90.3	86.7-93.8
Hand-foot-mouth disease	1,818,000	70.0	66.7-73.4
Erythema infectiosum	299,000	11.5	10.7-12.4
Exanthema subitum	1,084,000	41.8	39.8-43.7
Pertussis	28,000	1.1	1.0-1.2
Rubella	32,000	1.2	1.0-1.4
Herpangina	1,300,000	50.1	47.2-53.0
Measles	191,000	7.4	6.8-7.9
Mumps	1,141,000	44.0	41.4-46.5

The incidence rate and its 95% confidence interval are stated per 1,000 population aged 0-19 years.

Table 4 shows the numbers of all disease outpatients per day in all medical institutions and SMIs. For each type of medical institution, the mean number of all disease outpatients per day was higher in SMIs than in all medical institutions.

**Table 4.** The numbers of all disease outpatients per day in all and sentinel medical institutions.

	Department	All medical institutions			Sentinel medical institutions					

					for Influenza-like illness			for Pediatric diseases		
		
		No.	Mean	SD	No.	Mean	SD	No.	Mean	SD
Hospital	Internal Medicine	8,130	94.9	117.7	456	185.1	139.2			
	Pediatrics	3,575	34.2	40.5	726	66.0	43.3	730	65.8	43.3
Clinic	Internal Medicine only	37,454	37.0	61.5	470	70.3	50.2			
	Pediatrics only	3,411	45.1	38.9	1,155	53.5	34.9	1,145	53.9	35.5
	Internal Medicine and Pediatrics	23,842	44.6	49.7	1,778	62.5	44.1	1,116	60.8	44.1

SD: standard deviation

Table 5 shows the incidences of influenza-like illness and 12 pediatric diseases estimated from the hypothetical incidences in all SMIs, and their hypothetical true ones. The ratio of the estimate to the hypothetical true one was 1.00-1.09 among influenza-like illness and 2 pediatric diseases, 1.10-1.19 among 8 pediatric diseases, and 1.20-1.29 among 2 pediatric diseases (pertussis and measles).

**Table 5.** Estimated and hypothetical true incidences of influenza-like illness and pediatric diseases.

Disease	Hypothetical true incidence (a)	Estimated incidence (b)	Ratio (b/a)
Influenza-like illness	9,110,000	9,680,000	1.06
Pharyngoconjunctival fever	153,000	169,000	1.10
Group A streptococcal pharyngitis	1,075,000	1,279,000	1.19
Infectious gastroenteritis	6,788,000	7,403,000	1.09
Chickenpox	2,058,000	2,345,000	1.14
Hand-foot-mouth disease	1,612,000	1,820,000	1.13
Erythema infectiosum	275,000	300,000	1.09
Exanthema subitum	967,000	1,084,000	1.12
Pertussis	22,000	28,000	1.24
Rubella	28,000	32,000	1.12
Herpangina	1,148,000	1,300,000	1.13
Measles	152,000	191,000	1.26
Mumps	1,015,000	1,139,000	1.12

The estimated incidence was obtained from hypothetical incidences in all SMIs.

## DISCUSSION

The incidence rates of influenza-like illness and pediatric diseases in 2000 in Japan were estimated from surveillance data under the assumption that SMIs were randomly selected from all medical institutions. No information strictly comparable with our results was available because the incidence rates of infectious diseases varied widely among years and areas. Compared among the weekly numbers of patients per SMI in 1990-2000 in Japan (http://idsc.nih.go.jp/index.html), the epidemic in 2000 was large in group A streptococcal pharyngitis, pertussis and herpangina, small in rubella, and moderate in influenza-like illness, chickenpox and other diseases. The estimated incidence rate of influenza-like illness in 2000 in Japan (75.6 per 1,000 population) was higher than the figures reported in United Kingdom and France (16 and 42 per 1,000 population in the epidemic period of 1993 and 1995/1996, respectively),^14^^,^^18^ and within the range reported in The Netherlands (15-86 per 1,000 population between 1971-1989).^3^ The finding in this study that the incidence rate of influenza-like illness decreased with age, has been already reported.^14^^,^^19^ The incidence rate of chickenpox, as an example of pediatric diseases, is to be compared with those among several countries. The estimate in this study (90.3 per 1,000 population aged 0-19 years) was converted into about 18 per 1,000 population of all ages because the incidence rate of chickenpox was very low in population aged 20 years or over. This figure was higher than reported in UK and France (2-9 and 10-13 per 1,000 population in 1967-1985 and 1991-1995, respectively).^20^^,^^21^

The assumption of random selection of SMIs was critical.^11^ We attempted to evaluate possible bias in the estimated incidence using the number of all disease outpatients per day. The mean number of all disease outpatients per day was higher in SMIs than in all medical institutions. Although the number of all disease outpatients per day would not be the best index to the underlying SMI population, especially in infectious diseases with a great seasonal variation such as influenza-like illness, it suggested that the mean size of the underlying population was larger in SMIs than in all medical institutions, and that the assumption of random selection of SMIs would not be strictly valid. Since this assumption was undermined, the incidence rates of infectious diseases were overestimated. The ratio of the estimated incidence to the hypothetical true one was calculated to be 1.06-1.26 among infectious diseases and to be over 1.20 in pertussis and measles. However, the ratio would not be sufficiently accurate to be used for adjusting the estimated incidence rate. In the infectious diseases in which the ratio was less than 1.1 or 1.2, the estimated incidence rate might be useful for public health activities. The estimated incidence rate and its ratio to the hypothetical true one, even though not strictly accurate, provide some helpful information for planning vaccinations against infectious diseases such as influenza and rubella.^4^^,^^19^

In this study, we encountered several further problems and limitations in estimating the incidence rate from sentinel surveillance data. Although the criteria for diagnosis of infectious diseases were determined in the guidelines by the Ministry of Health, Labor and Welfare of Japan,^9^^,^^15^ the reports from SMIs might not always be based on such criteria. The incidence estimated in this study did not include persons with infectious diseases who had not visited medical institutions because information was available only from medical institutions. This limitation should be kept in mind when regarding the figures. Prefectures and types of medical institution were used as the strata in the method for estimating incidence rates. The assumption in the method was that SMIs were randomly selected in each stratum, not in all medical institutions. The strata used would be only natural because prefectures selected SMIs. The underlying population and patient characteristics such as age would differ greatly between hospitals and clinics and among departments as well. As described above, departments of internal medicine in hospitals and clinics with only a department of internal medicine were combined as to the type of medical institution for estimating the incidence rate of influenza-like illness because the number of SMIs in either the departments of internal medicine in hospitals or the clinics with only a department of internal medicine was 1 or less in several prefectures.

We think that the incidence rates of infectious diseases would not be completely accurate when estimated by only the information obtained from routine sentinel surveillance. However, these estimates, albeit somewhat biased, would still be useful for public health activities. Further research of methods for estimating incidence rates from sentinel surveillance data and evaluating their biases is important.

## APPENDIX

Consider the distribution of incidences in medical institutions. Let *m* be an integer greater than the largest incidence among medical institutions, *n* be the number of all medical institutions, and *n_i_* be the number of medical institutions with the incidence of *i* for *i* =0, 1, …, *m*. Let *N* be the number of SMIs, and *N_i_* be the number of SMIs with the incidence of *i* for *i* =0, 1, …, *m*. The constants of *n* and *N* are known, and those of {*n_i_*} are unknown. {*N_i_*} are obtained from the sentinel surveillance, and follow a multi-hypergeometric distribution under the condition of *N* fixed under the assumption that SMIs are randomly selected in all medical institutions.

Let *a* be the total incidence in all medical institutions, and note that *a*= Σ *i* **n_i_*. The estimate of *a* is given to be *a*^= Σ *i* **Ni***n*/*N*, i.e., the incidence is estimated as the total incidence in SMIs (Σ *i* **N_i_*) divided by the proportion of SMIs among all medical institutions (*N*/*n*).

The approximate confidence interval for *a* is given to be (*a*^-1.96**s*, *a*^+1.96**s*), where *s*^2^ is an estimate of variance of *a*^ and is given to be

{Σ *i*
^2^**Ni*/*N*-(Σ *i* **Ni*/*N*)^2^}**n*^3^(1/*N*-1/*n*)/(*n*-1).

Consider that the incidences in some strata such as prefectures and types of medical institution are estimated using the above-explained method. Let *k* be the number of strata, *a*_1_^, *a*_2_^, …, *a*_k_^ the estimated incidences in the strata, and *s*_1_^2^, *s*_2_^2^, …, *s*_k_^2^ their estimated variances. The approximate confidence interval for the total incidence is given as (*a*_t_^-1.96**s*_t_, *a*_t_^+l.96**s*_t_), where *a*_t_^=*a*_1_^+*a*_2_^+…+*a*_k_^ and *s*_t_^2^=*s*_1_^2^+*s*_2_^2^+…+*s*_k_^2^.
